# Revision for periprosthetic joint infection rate stratified by seasonality of operation in a national population of total and unicompartmental knee arthroplasty patients: a register-based analysis

**DOI:** 10.5194/jbji-6-111-2021

**Published:** 2021-03-05

**Authors:** Julius Tetens Hald, Anne Brun Hesselvig, Andreas Kryger Jensen, Anders Odgaard

**Affiliations:** 1 Department of Orthopedic Surgery, Copenhagen University Hospital Herlev-Gentofte, Kildegårdsvej 28, 2900 Hellerup, Denmark; 2 Department of Clinical Microbiology, Copenhagen University Hospital, Rigshospitalet, Henrik Harpestrengsvej 4A, 2100 Copenhagen Ø, Denmark; 3 Section of Biostatistics, Department of Public Health, University of Copenhagen, Øster Farimagsgade 5, 1014 Copenhagen K, Denmark; 4 Department of Orthopaedic Surgery, Rigshospitalet Copenhagen University Hospital, Blegdamsvej 9, 2100 Copenhagen Ø, Denmark; 5 Department of Clinical Medicine, University of Copenhagen, Copenhagen, Denmark

## Abstract

**Aim**: The aim of this study was to investigate whether the revision rate for periprosthetic joint infection (PJI) depends on the season of the primary procedure using a national population of knee arthroplasty (KA) patients. Seasonal variation of some surgical procedures has been observed to impact subsequent infection risks, with a higher risk of revision for surgeries performed during summer, but an analysis of PJI rates based on a national arthroplasty register has yet to be completed. We hypothesized that an increased risk of revision due to PJI could be demonstrated in a national population when primary surgery was performed during the summer. **Methods**: The Danish Knee Arthroplasty Registry (DKR) was used to determine the risk of revision due to PJI within 2 years after primary surgery. All primary KA procedures between 1 January 1997 and 31 December 2014 and revisions until 31 December 2016 were identified. Smoothing spline regression was used to identify possible seasonal pattern effects of the primary procedure on revision risk, and logistic regression was used to calculate risk of infection differences between seasons. **Results**: A total number of 124 809 primary procedures was registered in the study period. After excluding duplicates and matching primary procedures with the first revisions within 2 years after the primary procedure, 3391 were identified. Of these, 348 cases were recorded with an indication of deep infection requiring revision. Spline regression analyses did not demonstrate any clear seasonal pattern of the primary procedure regarding the risk of revision for infection or any other cause. Logistic regression found a decreased risk of revision for infection when the primary procedure was performed during the summer in the years 1997 to 2005, no influence on the risk of revision for infection in 2005 to 2012, and an increased risk of revision for infection following summer procedures during the years 2013 to 2014. **Conclusion**: It was not possible to demonstrate a consistent seasonal variation of the risk of revision for PJI following primary KA. This is most likely because the underlying etiologies for PJI are not subject to seasonal variation.

5 March 2021

## 1 Introduction

Knee arthroplasty (KA) is a frequent procedure used to treat knee osteoarthritis (OA) (Mahomed et al., 2005). Periprosthetic joint infection (PJI) is a serious complication and is associated with higher morbidity, mortality, increased hospitalization, and increased hospital expenditures (Daines et al., 2015; Garrido-Gomez et al., 2013). Infection prevention is therefore a constant subject of interest in orthopedic research. Several risk factors for PJI following primary KA have been identified: diabetes mellitus, obesity, male sex, wound-related complications, duration of surgery, and rheumatoid arthritis (Jamsen et al., 2009; Daines et al., 2015; Cordtz et al., 2018; Badawy et al., 2017). The present study's authors hypothesized that a seasonal variation might also impact the rate of PJIs reflected in the rate of primary revisions.

Seasonal variation of surgical-site infections (SSIs) due to *Staphylococcus aureus* bacteria has been documented, and the highest risk of infection is during the summer (Leekha et al., 2012; Anthony et al., 2018, 2017; Durkin et al., 2015a). Higher temperatures, higher humidity, and increased sweating promote bacterial colonization on the human skin and may account for the increased infection rate (Leekha et al., 2012; Anthony et al., 2017, 2018). The seasonal variation of *S. aureus* is of interest as *S. aureus* is a common etiology for PJI in knee arthroplasty patients (Garvin and Konigsberg, 2011; Bengtson and Knutson, 1991; Leekha et al., 2012). Seasonal variation of postoperative infection rates has been observed in studies investigating SSIs after spine surgery and arthroplasty (Anthony et al., 2017, 2018; Durkin et al., 2015a, b; Grassly and Fraser, 2006; Gruskay et al., 2013; Kane et al., 2014; Leekha et al., 2012; Rosas et al., 2017; Sodhi et al., 2018). Anthony et al. (2017) found that patients who received surgery when the temperature was over 32 ${}^{\circ }$C had a 28.9 % increased risk of SSI admission (95 % CI: [20.2–38.3]) compared to patients who received surgery when the temperature was below 4 ${}^{\circ }$C (Anthony et al., 2017). Sodhi et al. (2018) investigated the influence of the season of primary TKA and the rate of 30 d postoperative complications. They found increased risk of superficial and deep infection following TKA when comparing the months July to September with January to March. These data came from hospitals located in every state in the USA (Sodhi et al., 2018). We believe some cases of deep infections might have been lost in the previous studies because some patients will be revised due to deep infection more than 30 d following the primary procedure. Finally, the abovementioned studies were conducted in climates that are very different from that of northern Europe (Sodhi et al., 2018; Kane et al., 2014; Anthony et al., 2017, 2018).

The present study investigates whether the risk of deep infection following KA shows a seasonal variation of the primary procedure in a temperate climate. We also examined a possible seasonal effect of the primary procedure on the overall revision rate. Our analysis is based on determining a periodical pattern in the risk of revision over time, and, if a pattern is found, then subsequently to determine the odds ratio (OR) associated with the season of primary surgery.

## 2 Methods

### 2.1 Data collection

The study was approved by the Danish Data Protection Agency (file number: BFH-2017-033). All data were acquired from The Danish Knee Arthroplasty Register (DKR) (2019). The DKR is a clinical database that has existed since 1 January 1997. Mandatory registration of all knee arthroplasties performed in Denmark has existed since 1 June 2006 (Pedersen et al., 2012). Every primary and revision arthroplasty is registered in detail and linked to each patient's unique identification number from the Danish Civil Registration System. We searched the DKR for all patients who between 1 January 1997 and 31 December 2014 had undergone primary KA resulting in revision surgery within 2 years. The DKR was then searched for all revision surgeries performed within this time frame with a follow-up period of 2 years after primary surgery. All duplicate registrations and consecutive revision procedures on the same knee beyond the first were excluded. Revisions were matched with the primary procedure registered with the same patient identification number and laterality of procedure. The date of both primary and revision surgery for each patient was extracted. Procedures without a registered date were excluded. Revisions due to infection were identified in the following manner. From 1 January 1997 to 31 December 2011, our criterion for a revision due to infection was the explicit classification of the surgery as due to “deep infection” by the operating surgeon. From 1 January 2012 and onwards, the surgeon would classify the revision as either “verified deep infection” or “suspected deep infection”. Both groups were included in our study population.

**Figure 1:**
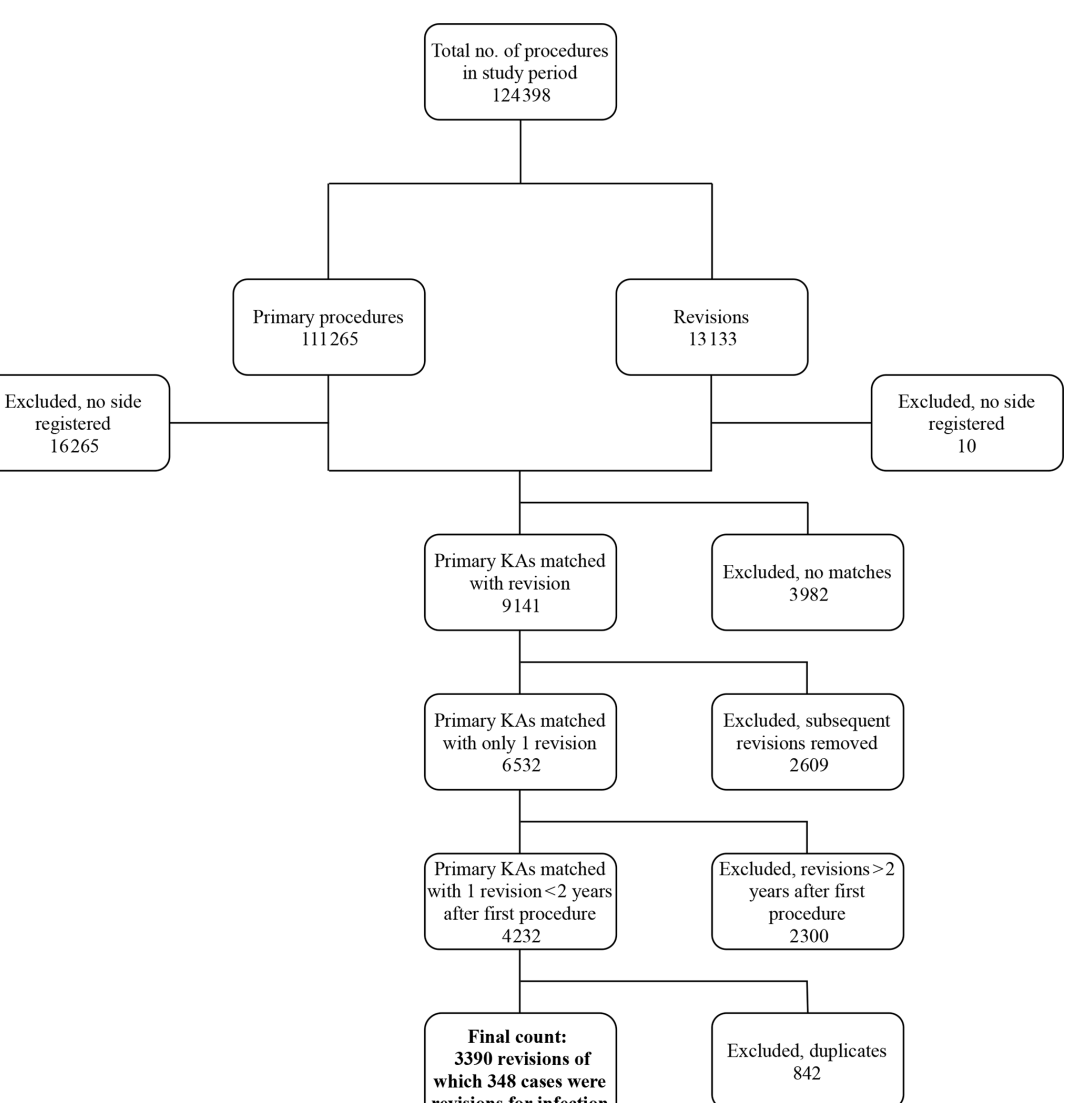
Flowchart describing the data-collection process.

### 2.2 Statistical approach

To investigate seasonal trends, we first calculated the empirical risks for each primary procedure at every month averaged across the years of (1) revision due to infection and (2) revision for any reason, both within 2 years after primary surgery. To flexibly model a periodic seasonal trend within a year while allowing it to change across years, we used logistic regression with penalized smoothing splines as covariates. The smoothing splines were formed as a full tensor product of main effects and the interaction of a slow-varying thin plate regression spline across calendar time and a cyclic cubic regression spline across the 12 months of each year (Wood, 2017). We also fitted a simplified model with parameters interpretable as odds ratios through a logistic regression with a linear effect of year as a continuous covariate, a dichotomization of the months into two seasons, May to August vs. September to April, and their interaction. All cases of revisions were assigned to one of the two seasons, based on the month of the primary procedure. Statistical analyses were performed using R (R Core Team, 2020).

## 3 Results

Data were gathered on a total of 124 398 procedures. Figure 1 shows the data-collection process. The number of primary procedures and revisions during the 18 years was 111 265 and 13 133, respectively. Primary procedures were matched with revisions, resulting in 9141 cases. After excluding subsequent revisions (n=2609), the number of primary revisions was 6532. We then excluded 2300 revisions because they were performed >2 years after primary surgery. After excluding duplicate registrations, 3390 revisions for any reason were identified; 348 of the 3390 cases were revisions for infection. Data were separated into two groups: first, those which were revised due to deep infection within 2 years of primary surgery, and second, those which were revised for any reason within 2 years of primary surgery.

**Figure 2:**
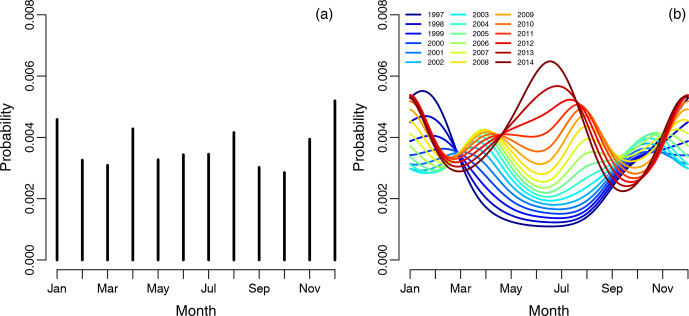
**(a)** Graph showing the empirical risk of revision for infection associated with each month and averaged over the years. Each vertical line represents 1 calendar month, with risk of revision due to infection as the dependent variable. Panel **(b)** shows the spline regression model, accounting for month-by-year interaction on the risk of infection. Each curve represents 1 year in the study period.

### 3.1 Revision due to infection within 2 years of primary surgery

The empirical risk of revision of infection associated with each month and averaged over the years did not show any obvious periodical pattern shown in Fig. 2a. The spline regression model, accounting for season-by-year interaction on the risk of infection revision, is shown in Fig. 2b, illustrating that the risk of revision of infection has changed dramatically over the years. The risk of revision of infection is lowest when primary surgery is performed during the summers of 1997 to 2006. Between 2006 and 2012 there is no obvious difference in the risk of revision due to infection when comparing the summer months to the winter months, but the risk of revision due to infection in summer months increases over time. During the summers of 2013 to 2014, there is a clear peak in revision due to infection when the primary procedure was performed during the summer and winter.

**Figure 3:**
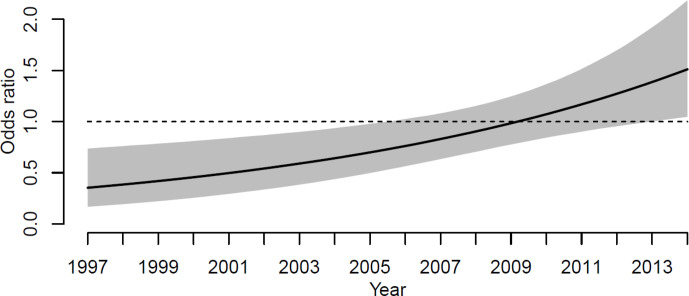
The odds ratio for revision due to infection when comparing the seasons for primary procedure (May to August vs. September to April). The line shows the odds ratio and the gray region its corresponding point-wise 95 % confidence interval.

Subsequently, logistic regression was used to quantify any differences in the risks of revision for infection between the two seasons, May to August and September to April. The risk of revision due to infection between the two seasons changed during the study period. Figure 3 shows the odds ratio for revision when surgery was performed during the 4 summer months compared to the rest of the year. The odds ratio of revision for infection was significantly lower for primary KA performed during the summers of 1997 to 2006. In 1997, the odds ratio is 0.4 (CI 95 %, 0.2–0.7), and in 2006 the odds ratio is 0.7 (CI 95 %, 0.5–1.0). From 2006 to 2013 the odds ratio for revision for infection rises, but the odds ratio is not significant. From 2013 to 2014 the odds ratio of revision for infection is significantly higher when the primary procedure was performed in the summer. In 2014 the odds ratio was 1.5 (1.01–2.1, 95 % CI).

### 3.2 Revisions within 2 years due to any reason following primary surgery

**Figure 4:**
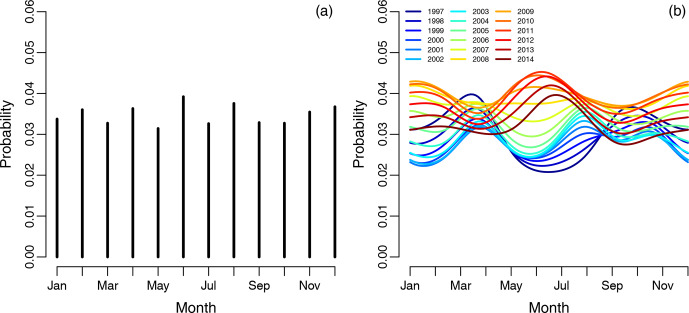
**(a)** The empirical risk of revision for any reason for each month averaged over the years. Each line represents 1 calendar month. **(b)** The spline regression model showing the probability of revision for any reason.

The empirical risk of revision for any reason associated with each month and averaged over the years did not show any obvious periodical pattern shown in Fig. 4a, as for infections. The risk of revision was lowest when primary surgery was done during the summer in the period 1997 to 2006, shown on the spline regression model in Fig. 4b. The risk of revision is somewhat inconclusive from 2006 to 2012, but it is obvious that the revision risk is increasing during the study period when the primary procedure is performed during summer. The risk of revision was highest when primary surgery was done during the summer in the period 2013 to 2014, but there is also a noticeable peak during the winter months.

## 4 Discussion

The results of this study failed to demonstrate that a general seasonal variation of the primary KA procedure risk of subsequent revision of infection or due to other factors is present in the temperate climate of Denmark. For the revision for infection risk, we found an increased risk when primary KA was performed during the winter, from 1997 to 2006. From 2006, the results are inconclusive, but in 2013 a significantly increased risk of revision for infection was found when the primary procedure was performed during the summer season. The summer season evolves from protecting against revision due to infection to no influence on revision for infection risk and then finally to be a risk factor for revision for infection. Similar results were derived when looking at the overall revision rate. No consistent periodical pattern was found.

The most likely explanation for our finding is that the risk factors of PJIs are not subject to seasonal variation, unlike SSI rates, which have been shown to be associated with warmer temperatures (Leekha et al., 2012; Anthony et al., 2017, 2018; Kane et al., 2014). SSIs include infections of the skin, suture wounds, and superficial soft tissue as well as deep infections. Increased bacterial load on the skin during summer could explain the relationship between the summer season and the increased rate of SSIs. However, this may not impact the PJI rate because PJI is a deep infection in the tissue surrounding the prosthesis. Bacteria are less likely to reach the joint or prosthesis, in contrast to the aforementioned superficial structures. Also, these studies were conducted in warmer climates. In addition, PJI is probably caused by a variety of reasons, often multifactorial, such as a mix of the patient's disposing factors, surgical technique, type of prosthesis, and antibacterial prophylaxis. All these factors could contribute to the different seasonal patterns that were discernible throughout the study period. Randomness of incidence of infections could influence our findings. The absolute incidence of PJI following KA rose slightly from 1997 to 2010, whereafter it remained constant. The relative incidence of infection varied from 0.2 % to 0.6 % during the study period, with no seasonal differences. This finding does not explain the different seasonal patterns which we found. The protocol for KA has changed in some aspects over the study period of 17 years, such as a marked reduction of length of hospital stay, decreased use of drain postoperatively, and increased focus on general infection-prevention measures. However, it is highly unlikely that any of these factors, which affect the postoperative course following KA, would influence or be subject to any seasonal variation.

Our results show that the PJI rate following primary procedures during the summer months increased over the study period. The average temperature in Denmark for the 4-month period of May to August in 2001 was 14.6 ${}^{\circ }$C, no clear change was observed in the following 14 years, and year-to-year variations were below 1 ${}^{\circ }$C. The seasonal variation that has been found in previous studies could not be found in our study, which may be caused by the climate differences between the previous studies' origin and northern Europe (Leekha et al., 2012; Anthony et al., 2017, 2018; Kane et al., 2014). If an increased risk of PJI exists with higher temperatures, the increase in average temperature could in the future highlight a weak seasonal periodicity in the rate of infections.

### Limitations

A possible limitation of our study is an incomplete DKR registration rate. An incomplete registration rate could explain the differences in seasonal patterns in our results. However, it is unlikely that a systematic bias would exist, and it can reasonably be assumed that missed registrations would be evenly distributed. Also, the completeness rate from the DKR is high: in 2001 it was 75 %, and it peaked at 97 % in 2014, meaning that only very few registrations were missed (The Danish Knee Arthroplasty Register, 2019).

Our findings were partly based on the surgeon's ability to correctly identify PJI. It is not easy to differentiate between aseptic loosening and subclinical PJI with culture-negative aspirations, and some cases of aseptic failures may have been due to occult infection (Parvizi et al., 2011). We included our analysis of the revision for any reason within 2 years of primary surgery, in order to include late infections. Revisions within 2 years of primary surgery are due to serious complications, which develop rapidly. An occult infection in the periprosthetic tissue that is not detected can be mistaken for another complication, which justifies revision. Also, studies have found that surgeons are prone to underreporting PJIs, because PJIs are often diagnosed perioperatively or within 30 d after primary procedure (Gundtoft et al., 2015, 2016). A better registration of PJIs is possible by merging data from multiple databases, preferably data from a microbiological database, as demonstrated by Gundtoft et al. (2015, 2016), who found a significantly increased rate of PJIs when merging the Danish Hip Arthroplasty Register with several, including microbiological, databases. Because PJIs may have been underreported, we performed an analysis of the 2-year revision for any reason rate so as to include missed infections, but as shown, this did not demonstrate any periodic pattern. The present study did not have access to microbiological data. Including all pathogens, as we did in our study, could suppress a signal of seasonal variability of a single pathogen, for example, *S. aureus*. *S. aureus* being more virulent, this pathogen is likely to be responsible for the acute/early infection cases. We encourage future studies to include microbiological data, because some pathogens may display seasonal variation, but others may not.

## 5 Conclusion

We did not find any definite periodical signal indicating a seasonal variation of the PJI rate. There may be several reasons for our findings, but the most likely is that there is no association between PJI and season in temperate climates. It is clear, from previous studies, that an association between warm weather and SSIs exists. We did find an increased risk of infection, when the primary procedure is performed during the summer, in the last 2 years of the study period. As temperatures continue to rise, a weak association between higher temperatures and the PJI rate may become evident in the future.

## Data Availability

No code or data could be stored in any databases because data contain personally identifiable information. Storing this information in unauthorized databases is not allowed by the Danish Data Protection Agency.
